# Estimating herbarium specimen digitization rates: Accounting for human experience

**DOI:** 10.1002/aps3.11415

**Published:** 2021-04-30

**Authors:** Caleb Powell, Alaina Krakowiak, Rachel Fuller, Erica Rylander, Emily Gillespie, Shawn Krosnick, Brad Ruhfel, Ashley B. Morris, Joey Shaw

**Affiliations:** ^1^ Department of Biology, Geology, and Environmental Science University of Tennessee at Chattanooga 615 McCallie Avenue Chattanooga Tennessee 37403 USA; ^2^ Department of Biological Sciences Butler University 4600 Sunset Avenue Indianapolis Indiana 46208 USA; ^3^ Department of Biology Tennessee Tech University 1 William L. Jones Drive Cookeville Tennessee 38505 USA; ^4^ University of Michigan Herbarium University of Michigan 3600 Varsity Drive Ann Arbor Michigan 48108 USA; ^5^ Department of Biology Furman University 3300 Poinsett Highway Greenville South Carolina 29613 USA

**Keywords:** biodiversity data, digitization rates, herbaria, natural history collections

## Abstract

**Premise:**

Herbaria are invaluable sources for understanding the natural world, and in recent years there has been a concerted effort to digitize these collections. To organize such efforts, a method for estimating the necessary labor is desired. This work analyzes digitization productivity reports of 105 participants from eight herbaria, deriving generalized labor estimates that account for human experience.

**Methods and Results:**

Individuals’ rates of digitization were grouped based on cumulative time performing each task and then used to estimate a series of generalized labor projection models. In most cases, productivity was shown to improve with experience, suggesting longer technician retention can reduce labor requirements by 20%.

**Conclusions:**

Using student labor is a common tactic for digitization efforts, and the resulting outreach exposes future professionals to natural history collections. However, overcoming the learning curve should be considered when estimating the labor necessary to digitize a collection.

During the digitization of eight herbaria (Table 1) affiliated with the SouthEast Regional Network of Expertise and Collections (SERNEC), task‐specific data were collected over 34 months (June 2016 to May 2019) spanning 7808 hours across a workforce of 105 people producing more than 273,000 digitized herbarium specimen records. The labor costs associated with these efforts consumed the majority of their respective budgets (>96%). Here, an analysis of these data is presented as a reference for planning future digitization efforts. Most of the technicians involved in these efforts were undergraduate students, many of whom (including four of the authors of this manuscript) were first introduced to natural history collections through these initiatives. It is therefore presumed that the average participant of this study had little or no pre‐existing expertise in natural history collections. In general, the rate of manual task performance is expected to improve as the operator gains experience in the task (Motowidlo and Van Scotter, 1994). Consequently, future digitization efforts planning to use inexperienced technicians may benefit from accounting for changing rates of performance as well as the longer technician retention times associated with larger specimen counts.

**Table 1:** The collections affiliated with this study and their contributions. Factors that may contribute to unequal specimen counts are: incomplete reporting, data cleaning, or workflow differences such as existing progress from previous efforts.

Collection	Collection code	Participants	Total hours	Barcode application (Specimens)	Imaging (Specimens)	Skeletal data entry (Specimens)
Berea College, Ralph L. Thompson Herbarium	BEREA	7	237	51	22,754	22,105
East Tennessee State University	ETSU	7	127	9861	9841	4355
Middle Tennessee State University	MTSU	17	875	3348	19,438	73,639
Rhodes College	SWMT	5	174	4881	5473	15,789
Tennessee Technological University	HTTU	25	520	18,053	12,658	15,950
University of Tennessee at Chattanooga	UCHT	42	1004	51,351	39,997	34,478
University of Tennessee, Knoxville	TENN	36	2960	174,118	195,797	175,966
University of Tennessee at Martin	UTM	2	49	1810	2150	2736
University of the South	UOS	3	8	525	423	661

Factors such as location, space, specimen organization, and scope of digitization tasks make every digitization effort unique and difficult to generalize. Due to this uncertainty, the pre‐digitization curation necessary to prepare for such a project (e.g., specimen retrieval, organization, and annotation) is not examined in detail. Tasks that ultimately follow pre‐digitization curation (i.e., “digitization tasks”) include: affixing barcode labels (referred to herein as “barcoding”), imaging, and transcribing label data (either complete specimen label data, which we refer to as “complete databasing,” or a minimal subset of label data, referred to herein as “skeletal databasing”).

Few works have published digitization rates from which to reference (Nelson et al., 2012; Tulig et al., 2012; Harris and Marsico, 2017; Sweeney et al., 2018), and some of these are published as combined task rates. This limited availability of task‐specific data represents a challenge for future digitization projects seeking reference task rates. Tulig et al. (2012) presented complete databasing rates of 0.167 specimens per minute (SPM) and skeletal rates of 2.083 SPM. In both cases, these rates included barcode label application. Additionally, Tulig et al. (2012) reported an image capture rate of 1.417 exposures per minute (EPM), a metric distinct from SPM in that it accounts for the infrequent incidence of a single specimen occupying multiple herbarium sheets. Nelson et al. (2012) and Thiers et al. (2016) both presented an imaging capture rate of approximately 1.667 herbarium sheets per minute, which is functionally equivalent to EPM (Nelson et al., 2012). Harris and Marsico (2017) published a complete databasing rate of 0.417 SPM based on undergraduate student averages as well as one graduate student’s rate of 0.783 SPM. In the same work, the authors also presented a combined average imaging rate of 2.417 SPM. Sweeney et al. (2018) published digitization rates resulting from an automated conveyor system, which combined imaging and data capture of a set of fields significantly exceeding skeletal databasing at a rate of 0.593 SPM when accounting for system minutes, and 0.375 SPM when accounting for combined operator minutes (i.e., “person minutes”). Additionally, Sweeney et al. (2018) presented an imaging‐only rate of 2.19 SPM when accounting for system minutes. Although valuable references for planning a digitization effort, none of these works specifically accounts for the productivity increases associated with task mastery as a function of technician experience.

The data gathered and analyzed for the present study are highly granular, containing individual technician rates per task per work session. Using this level of specificity, we assessed the rate of task performance and provide guidance on estimating the labor required to digitize a herbarium. The objectives of this analysis were to: (1) determine the average rate of worker improvement as a function of experience, and (2) derive labor estimates across a range of specimen counts for barcoding, skeletal databasing, and imaging tasks. Additionally, we attempt to characterize the nature and impact of the unexpected setbacks experienced throughout the project.

## METHODS AND RESULTS

### Scope of digitization tasks

Technicians from each collection were trained on digitization tasks and provided stepwise workflows consistent with those proposed by Nelson et al. (2015). Training for each task typically involved one work session guided by an experienced peer or project leader. Training sessions lasted until technicians were able to perform the task without supervision, typically lasting between 30 minutes and two hours. All tasks included retrieving and replacing specimens from nearby cabinets (same room), and when possible, multiple folders were retrieved at once. During task performance, technicians were trained to preserve the order of the specimens and folders, simplifying specimen replacement. Skeletal databasing was performed directly in the SERNEC portal (https://sernecportal.org), which was built on the Symbiota platform (Gries et al., 2014), using the skeletal data entry tool and generic USB barcode scanners. Generally, the skeletal data transcribed in this data set included: a collection‐unique barcode number called a catalog number, the specimen’s scientific name, and the state and county in which the specimen was collected. Because Symbiota’s skeletal data entry tool automatically fills in authority and family data, these fields were captured but not transcribed. Barcoding involved applying archival stickers with unique identifiers to herbarium sheets in a consistent fashion. Imaging at each collection was performed using functionally similar sets of equipment, and in practice involved moving specimens from one stack into a closed light box system, capturing a photograph, and moving them to another stack. Initially, image files were named using camera controls as described by Nelson et al. (2015); however, shortly after the project began this was automated with custom barcode‐reading software (https://github.com/CapPow/bcAudit).

### Data collection

Google Forms was used as a reporting tool whereby any workers on this project, whether volunteers, hourly workers, work‐study students paid by a university, or students working for academic credit on independent study, were required to report minutes spent, tasks performed, and any setbacks experienced during each work session. For consistency, pulldown or selection menus were created for student name, date, and host herbarium. Additionally, data validation was enforced for numeric inputs (i.e., number of minutes performing a task and number of specimens on which the task was performed). Workers were instructed to never round time beyond the nearest five minutes and to report as precisely as possible the exact number of specimens barcoded, imaged, or skeletally databased. A free‐entry field was provided to describe any setbacks or problems that may have occurred that were not representative of a typical workflow (e.g., training, technical difficulties).

### Data cleaning

The task‐specific reports were cleaned and analyzed in Python 3.7 (https://www.python.org/), using the Pandas library (McKinney, 2010). Cleaning the data set omitted 2649 hours across 885 entries from the task rate analysis. Data set cleaning was based on the following criteria: non‐representative reporters (337 entries), exceptionally non‐representative workflow (3 entries), apparent entry errors (36 entries), indicated setbacks (453 entries), and extreme outliers (82 entries). Among the non‐representative reporters, one had additional technical and logistical responsibilities, three were discovered to be submitting fraudulent reports, and the remaining seven were suspected of inaccurate reporting (although not necessarily intentional). The 36 apparently erroneous entries were single reports that had such low task rates (specimens per minute) that the inverse ratio (minutes per specimens) was consistent with typical task averages. It is therefore assumed that data from those apparently erroneous entries may have been entered into the wrong fields.

The free‐entry field used for describing setbacks lacked data validation or controlled vocabulary and thus required explicit cleaning. All text in the setback descriptions had punctuation removed and was converted to lowercase. The most frequent words and phrases included among the setbacks were manually assessed to derive a set of acceptable phrases that indicate that no significant setbacks occurred (e.g., “no,” “none,” “no setbacks”). Any setback description not matching an acceptable phrase was identified as a non‐typical measurement resulting from a significant setback. These setback entries were set aside for separate evaluation and omitted from subsequent task rate analyses. Phrases indicating “training” were not among the acceptable phrases; therefore, the time invested during initial training of new technicians is not accounted for in the task rate analyses. Extreme outliers were defined as entries with any numeric values exceeding five standard deviations within that field. A total of 5158 hours across 2475 entries remained in the post‐cleaning data set following these cleaning operations.

### Data analysis

After cleaning, session report data were grouped by technician name and sorted by ascending date of work session. Additional fields were calculated to track individuals’ cumulative time performing each task at the time the report was submitted. All technicians’ cumulative task times were then grouped into shared two‐hour bins. The mean rate of performance (i.e., number of specimens per minute) for each task was calculated among all entries present in each two‐hour bin. This allowed non‐contemporary technicians to be compared at times when they had achieved approximately equal experience. The task rates per bin were then fit to a regression scatterplot using the Python Seaborn library (Waskom et al., 2018). A 64 cumulative‐hour threshold was selected for all tasks as the number of participants greatly diminished beyond that point. Imaging and skeletal databasing were fit to a simple linear model that was calculated using the linregress function available from the Python library Scipy (Virtanen et al., 2020). Barcoding was fit to a second‐order polynomial calculated using the polyfit function from the Python library Numpy (van der Walt et al., 2011).

**Figure 1:**
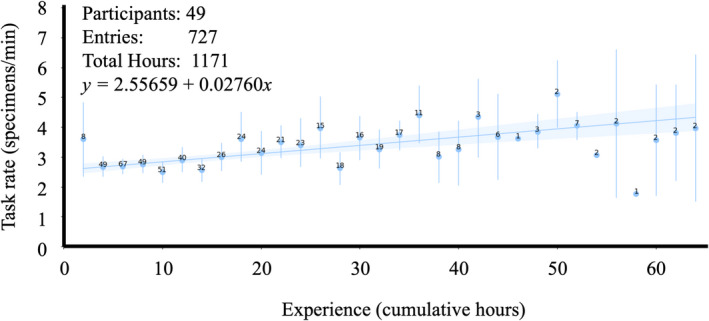
The average technician skeletal databasing rate (specimen/minute) as a function of cumulative hours performing skeletal databasing. The mean rate at each two‐hour bin is indicated by the blue point, and the number of data points informing the mean is annotated over each point. The range of values at each bin are indicated by vertical bars.

**Figure 2:**
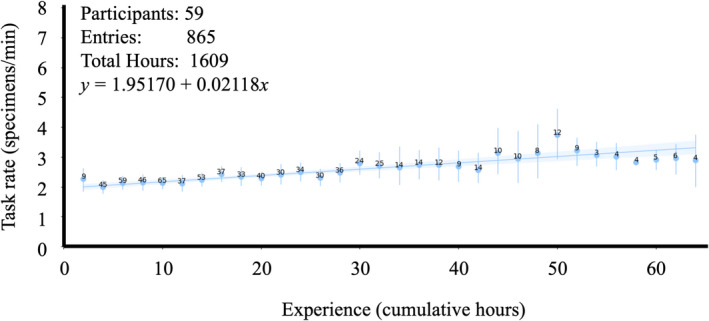
The average technician imaging rate (specimen/minute) as a function of cumulative hours imaging. The mean rate at each two‐hour bin is indicated by the blue point, and the number of data points informing the mean is annotated over each point. The range of values at each bin are indicated by vertical bars.

**Figure 3:**
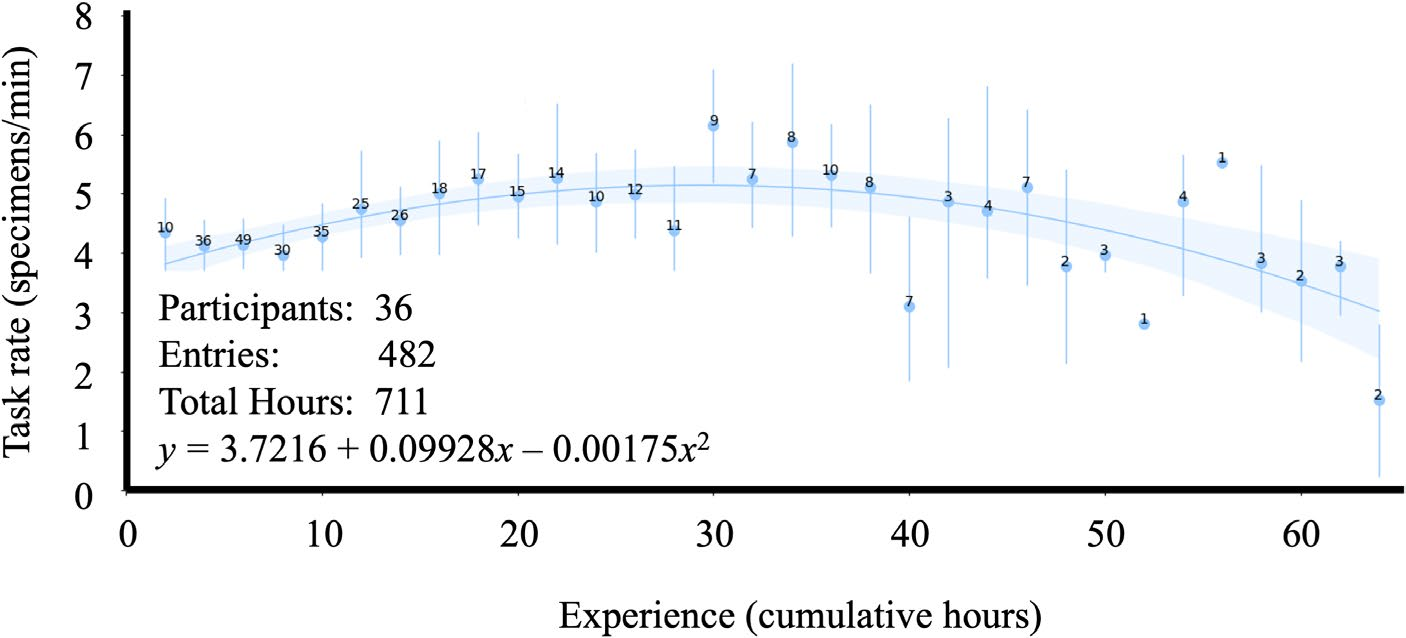
The average technician barcode application rate (specimen/minute) as a polynomial function of cumulative hours applying barcodes. The mean rate at each two‐hour bin is indicated by the blue point, and the number of data points informing the mean is annotated over each point. The range of values at each bin are indicated by vertical bars.

Using these models, a series of simulations was performed using Python to generate labor projections for digitization tasks as a function of technician turnover rate (Table 2). Each simulation accounted for technician turnover by resetting the task rate following the completion of a quantity of labor hours that represented the simulated technician’s contract duration (i.e., total time performing a single task) (Figs. 1, 2, 3). Contract durations simulated under these methods were: 15, 30, 45, 60, 90, and 135 hours performing each task. Because 90 and 135 cumulative hours for a single individual on a single task exceeds the 64‐hour model limits, rate limits were set based on extremes in the data set so that no rate estimations were extrapolated beyond the limits of the data set. Imaging, for example, was estimated using a linear model that produces higher rates as experience increases, yet at some point technical or physical limitations must impose a maximum possible rate. To avoid simulating unrealistically fast technicians, maximum rates of 4.00 SPM and 6.50 SPM were set for imaging and databasing, respectively, while a minimum rate of 3.00 SPM was set for barcode application. During simulations, rate estimates exceeding these limits were instead held at the respective limit.

**Table 2:** Labor estimates for digitization tasks for multiple specimen counts across multiple contract duration hours.

Contract duration (hours)	Task name	Specimen count												
		10,000	20,000	30,000	40,000	50,000	75,000	100,000	125,000	150,000	200,000	250,000	300,000	500,000
15	Barcoding	39	77	115	153	191	287	382	477	573	763	954	1145	1907
	Databasing	61	121	181	241	301	451	601	751	901	1201	1501	1801	3001
	Imaging	79	158	236	315	394	590	787	983	1179	1572	1965	2358	3929
	Combined	179	356	532	709	886	1328	1770	2211	2653	3536	4420	5304	8837
30	Barcoding	37	72	108	143	178	266	355	444	532	709	886	1063	1770
	Databasing	57	113	169	225	281	419	560	699	838	1118	1398	1676	2793
	Imaging	75	147	221	294	367	550	733	915	1098	1463	1828	2194	3656
	Combined	169	332	498	662	826	1235	1648	2058	2468	3290	4112	4933	8219
45	Barcoding	35	71	106	141	175	262	349	436	524	698	873	1047	1743
	Databasing	54	107	159	211	263	394	525	655	786	1047	1306	1568	2612
	Imaging	71	138	208	275	344	515	685	855	1027	1369	1709	2053	3418
	Combined	160	316	473	627	782	1171	1559	1946	2337	3114	3888	4668	7773
60	Barcoding	35	74	107	145	182	272	364	453	545	726	908	1089	1814
	Databasing	51	102	151	200	247	370	493	616	739	985	1230	1475	2454
	Imaging	66	131	196	260	325	482	645	806	964	1287	1607	1927	3212
	Combined	152	307	454	605	754	1124	1502	1875	2248	2998	3745	4491	7480
90	Barcoding	35	80	119	159	203	302	403	504	609	819	1020	1221	2040
	Databasing	51	90	141	180	231	341	450	567	680	899	1129	1348	2246
	Imaging	64	124	177	240	300	442	593	739	884	1176	1474	1767	2941
	Combined	150	294	437	579	734	1085	1446	1810	2173	2894	3623	4336	7227
135	Barcoding	35	80	135	170	214	323	440	558	673	887	1114	1346	2233
	Databasing	51	90	129	179	218	327	432	532	641	858	1064	1282	2127
	Imaging	64	114	175	227	281	422	561	701	840	1119	1397	1674	2782
	Combined	150	284	439	576	713	1072	1433	1791	2154	2864	3575	4302	7142

### Project setbacks

The 453 entries identified as significant setbacks totaled 946 hours, or 12% of total pre‐cleaning hours documented in this data set. Among those setbacks, 80 entries were identified as training sessions totaling 171 hours, or 2% of total pre‐cleaning hours. The mean duration of all sessions with setbacks was 133 minutes. By task, the mean duration of sessions with setbacks was 142 minutes for barcode application, 143 minutes for imaging, and 121 minutes for skeletal databasing. Conversely, the mean duration of all sessions without setbacks was 101 minutes. By task, the mean duration of sessions without setbacks was 88 minutes for barcode application, 112 minutes for imaging, and 97 minutes for skeletal databasing. Many of the most frequent words (and their frequencies) among setback descriptions were as follows: “specimen(s)” (68), “training” or “blitz” (59), “sernec” (43), “skeletal” (31), “slow” (25), “barcode(s)” (17), “imaging” (15), “folders” (14), “computer” (14), “label” (14), “missing” (13), “camera” (11), “collector” (11), “setting” (11), ”repair” (11), “internet” (9), “species” (9).

### Productivity: Project wide

Reporting a project‐wide digitization rate based on these data will depend on how one defines a specimen as being digitized and which hours are included as contributing to that effort. Here, three project‐wide rates are reported using various methods to determine both total specimens digitized and total hours contributing to the effort (Box 1). Using the pre‐cleaning average of each primary task’s total reported specimens as total specimens digitized and the sum of all pre‐cleaning hours reported across all tasks (Box 1A): all collections combined documented the digitization of 306,069 specimens at 0.653 per minute over a total of 7808 hours. Using the post‐cleaning average of each primary task’s total reported specimens as total specimens digitized and the sum of all post‐cleaning hours reported across all tasks (Box 1B): all collections combined documented the digitization of 213,863 specimens at 0.691 per minute over a total of 5158 hours. Finally, using the post‐cleaning average of each primary task’s total reported specimens as total specimens digitized and the post‐cleaning sum of only those hours spent on primary digitization tasks (Box 1C): all collections combined documented the digitization of 213,863 specimens at 0.983 per minute over a total of 3628 hours.

BOX 1 **Three methods used to calculate project‐wide digitization rates. (A) The calculation for specimen digitization rates that includes pre‐cleaned data reported for the three digitization tasks and pre‐cleaned data on labor minutes reported. (B) The calculation for digitization rates that includes post‐cleaned data reported for the three digitization tasks and all post‐cleaned labor minutes reported, including time not directly associated with the three digitization tasks. (C) The calculation for digitization rates that includes post‐cleaned data reported for the three digitization tasks and only post‐cleaned labor minutes reported that were associated with the three primary digitization tasks.**

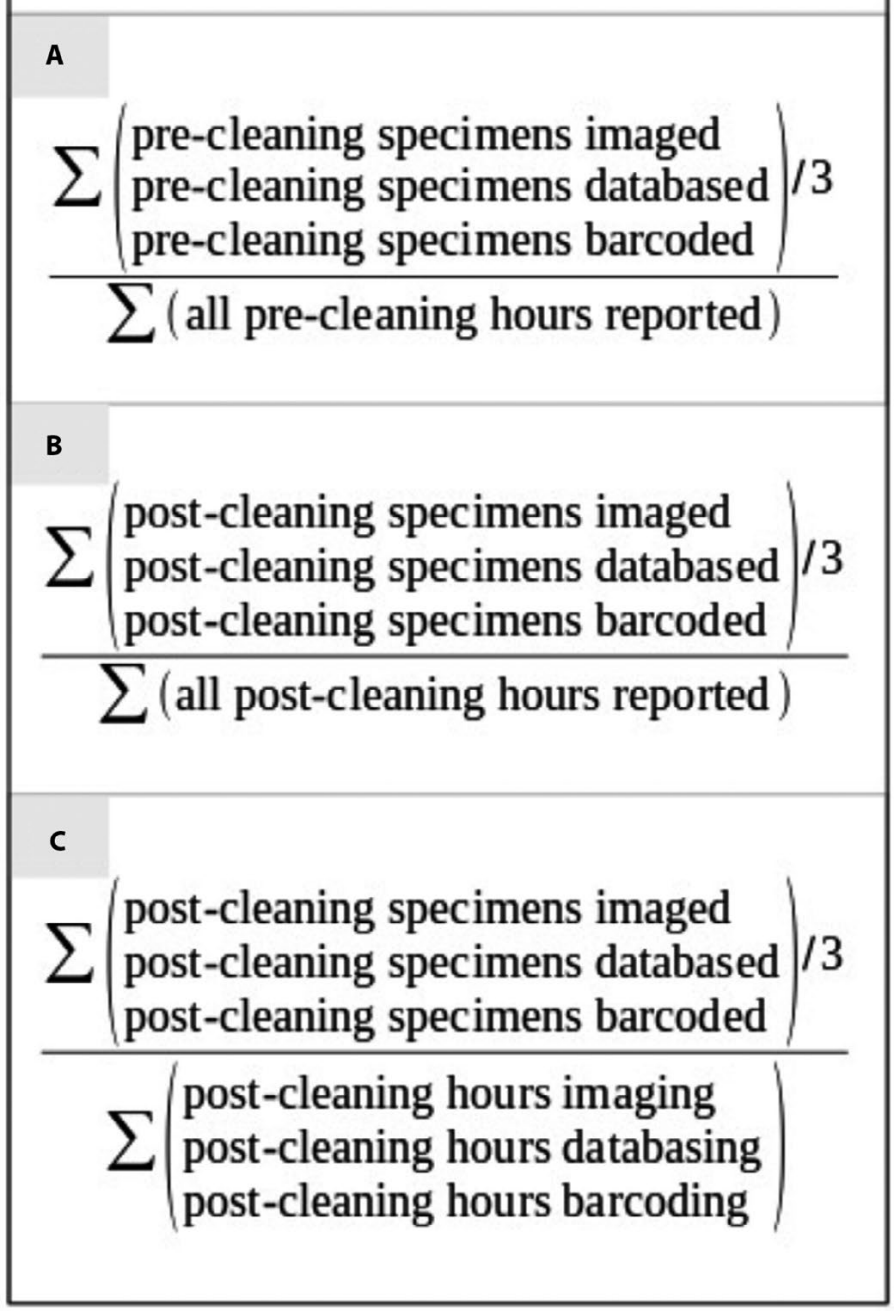

### Productivity: By task

Across all tasks and collections, 7808 hours were recorded; after cleaning, 5023 total hours remained. Of the post‐cleaning hours, 3493 were spent on the primary digitization tasks (i.e., imaging, skeletal databasing, barcode application) with the remaining 1530 hours spread across other tasks (e.g., pre‐digitization curation). Across all collections, post‐cleaning, primary digitization task rates were as follows: 229,333 specimens were imaged at 2.30 per minute over 1660 hours, while 231,307 specimens were skeletally databased at 3.14 per minute over 1228 hours, and 180,949 barcodes were applied at 4.07 per minute over 740 hours.

### Task rate estimations

Average imaging and databasing rates generally improved with technicians’ cumulative time performing the task and so were fit to linear functions (Figs. 1, 2). Imaging rate as a function of cumulative time is estimated as *y* = 1.95170 + 0.02118*x*. Skeletal databasing rate as a function of cumulative time is estimated as *y* = 2.55659 + 0.02760*x*. Although average barcode application rates initially improved with cumulative time, they exhibited an inflection point after which rates began to decrease following additional time performing the task. Therefore, barcode application rates were fit to a second‐order polynomial (Fig. 3). Barcode application rate as a function of cumulative time is estimated as *y* = 3.7216 + 0.09928*x* − 0.00175*x* ^2^. In each formula, *y* represents the estimated specimens per minute, whereas *x* represents the technician’s cumulative hours performing the specific task. The labor projections derived from these models are reported in Table 2.

## DISCUSSION

A priori planning and organization of a natural history digitization project, as well as individual technicians, will impact the efficiency of the tasks performed. Over the course of this work, technician imaging and databasing rates were shown to improve with experience (Figs. 1, 2). Barcode application rates, on the other hand, began to drop following 30 cumulative hours (Fig. 3). It was our assumption that this inflection point where barcoding rates begin to deteriorate reflects the ease of mastering the simple task and boredom with repetition. However, an alternative explanation could be technicians “graduating” to other tasks. Because barcode application was often the first task on which technicians were trained, it is possible that the observed degradation in mean barcode application rates is due to improving technicians progressing to more complex tasks, while those technicians exhibiting no improvements continued to barcode.

The labor estimates provided in Table 2 were calculated on a per‐task basis to facilitate different needs across various projects. Consequently, the contract duration used in Table 2 is task specific. A collection planning to utilize contract technicians at six hours a week for 15 weeks would only use a 90‐hour contract duration if those technicians always performed the same task. If instead those same technicians split their time across all three tasks, the appropriate per‐task contract duration would be 30 hours. Regardless of how workers are organized, the estimates suggest that high technician retention can reduce total labor requirements by up to 20%. Although high retention times detrimentally influence barcode rates, those rate losses are mitigated by the improvements achieved in the other tasks.

### The opportunity cost of efficiency

Although there are technical and physical limitations to the maximum achievable rates, technician retention time appears to influence overall digitization efficiency. In general, the longer technicians are engaged on a project, the more efficient the project will be. Longer retention times naturally imply fewer overall technicians. This intuitive outcome represents an unfortunate trade‐off between efficiency and the type of outreach that initially introduced many of the authors of this manuscript to this field. Integrating biological specimen digitization, or perhaps better yet, the use of digitized specimen data, into undergraduate coursework, may recoup the outreach opportunity cost associated with longer technician retention periods. Another solution to abate the reduction in outreach could be to use the barcoding task as an introduction while still maximizing retention times for those technicians performing imaging and databasing. This solution would have the additional benefit of utilizing the most efficient portions of each task’s performance curve (Figs. 1, 2, 3).

### Considerations when employing these estimations

This work is presented with the hope that it may be a useful reference for planning future digitization projects, yet we acknowledge two caveats that should be considered when using these data to formulate a labor budget. The first caveat is that the very process of collecting these data certainly has affected the results we present. During the 34‐month period of this work, technicians’ task‐specific reports became invaluable for planning and labor management. We believe the habit of reporting a task rate after each session helped maintain heightened awareness of individual and overall task rates. For example, these data were used to publicly praise individuals’ achievements either through team‐wide emails or in some cases, bi‐weekly leaderboards. Subjectively, it was observed that this heightened task rate awareness motivated many participants to improve over time by attempting to exceed their previous rates. We also observed that this awareness influenced individuals’ task selection with participants preferring the tasks in which they were most competitive. Additionally, actively monitoring individual reports helped team leaders identify and address areas of concern such as repeated setbacks and the aforementioned fraudulent reports. For these reasons, we believe that active management of the labor force based on these reported rates is integral to the rate improvements we documented in imaging and databasing.

The second caveat to consider is that by omitting extreme outliers, non‐representative reporters, and significant setbacks (including training), the derived estimation formulas assume unrealistically ideal scenarios. From the 7808 total pre‐cleaning hours documented, approximately 12% (946 hours) contained significant setbacks. The mean session duration (for all tasks combined) increased by 32% when a setback was present. Subjectively, the majority of the setbacks documented were of a technical nature (e.g., internet connectivity or camera settings). In addition to the setbacks, nearly 12% of pre‐cleaning hours (918 hours) were identified as having been from non‐representative reporters. Labor estimates derived from these formulas using 45‐hour contract durations underestimate the actual labor expenditures of the participating collections by 25%. In light of these caveats, we recommend efforts using these data include a similar session‐rate tracking system and add an appropriately sized “unanticipated setbacks” buffer (e.g., 20–30%) to the labor budget.

### Comparing these estimations to previously published rates

Labor estimations based on Table 2 or the formulas presented here were influenced by total specimen count, as well as expanding technician experience by way of contract duration. These estimates also assume a relatively similar workflow with respect to equipment, training, and specimen flow through the process. It is therefore difficult to formulate an equitable comparison among previously published rates. Among the recent works reporting digitization rates, the imaging process described by Harris and Marsico (2017) is similar to the workflow informing the estimations presented here. In their work, Harris and Marsico (2017) estimated that a single person could image 20,000 specimens in 13 10‐hour weeks (i.e., 130 hours). Our estimation methods assume technician turnover following specified contract durations; using an arbitrarily large contract duration therefore implies a single technician performing the task. In this way, we estimated one technician could image 20,000 specimens in 114 hours (Table 2). Our estimate of 114 hours falls 14.0% below that of Harris and Marsico’s (2017) estimated 130 hours and 20.1% below their observed rate of 2.417 SPM when extrapolated over 20,000 specimens (135 hours). Within the context of the caveats discussed above, these underestimates are anticipated. Tulig et al. (2012) presented a combined task rate for databasing partial records and barcoding of 2.083 SPM and 1.417 EPM for imaging. Extrapolating from their rate of 2.083 SPM suggests it would require 800 hours to barcode and skeletally database 100,000 specimens. Because the rates reported by Tulig et al. (2012) exclude technical training and troubleshooting, they are readily comparable to the estimation models presented here, assuming similar fields among the skeletal records we captured and the partial records they describe. Our estimates for performing these two tasks (i.e., barcoding and skeletal databasing) across 100,000 specimens ranged from 1388 hours using 15‐hour contracts to 872 hours using 135‐hour contracts. Given the scope of their work, we believe it is reasonable to assume that longer contract periods are more representative. Using 135‐hour contract durations, our methods overestimated the necessary hours reported by Tulig et al. (2012) by 9%. Extrapolating the 1.417 EPM imaging rate of Tulig et al. (2012) suggests it would require 1176 hours to image 100,000 specimens, whereas our imaging estimates range from 787 using 15‐hour contracts to 561 using 135‐hour contracts. Assuming longer contract periods and equivalency between SPM and EPM, our estimates are up to 52% lower than those based on the rates presented by Tulig et al. (2012). Increases in computational power and workflow differences may be contributing to this disparity. For example, the imaging process described by Tulig et al. (2012) included renaming image files using a barcode scanner, a step that was automated for the majority of the data informing our estimations.

## CONCLUSIONS

We evaluated 7808 hours of herbaria‐digitizing activities spanning 34 months across a workforce of 105 people. These data were assessed and presented in a way that highlights the average rate of worker improvement as a function of cumulative experience and to provide labor estimates for common digitization tasks; thus, they may be used by differently sized collections or projects to estimate labor costs. We believe the estimations presented are achievable when using similar workflows and incorporating individual session‐rate tracking tools into the digitization effort. Because these estimates represent the rates possible when no unforeseen delays are present, we recommend an additional 20–30% of labor funding be included to account for setbacks such as those discussed here.

## AUTHOR CONTRIBUTIONS

C.P. designed and performed the analysis, produced figures and tables, wrote the original draft, and assisted J.S. in data curation and project supervision. A.K., R.F., E.R., E.G., S.K., B.R., A.B.M., and J.S. substantially contributed to data curation, project administration, and supervision, as well as draft review and editing. A.B.M., J.S., E.G., and B.R. secured funding for the work. All authors approved the final version of the manuscript.

## Data Availability

Supplemental information including an anonymized form of the pre‐cleaning data and task‐specific labor estimation line graphs for multiple contract durations are available as a public GitHub repository (https://github.com/CapPow/digitization_rates_si).
